# Exploring the impact of ICT interaction on students’ creative thinking across performance levels: an explainable AI perspective

**DOI:** 10.3389/fpsyg.2025.1655731

**Published:** 2026-01-08

**Authors:** Xinhai Cai, Yi Bi, Yonggang Feng

**Affiliations:** 1 Faculty of Education, Shandong Normal University, Jinan, China; 2 Office of Development Planning and Discipline Construction, Yantai University, Yantai, China

**Keywords:** creative thinking, ICT interaction, explainable AI, SHAP, PISA 2022

## Abstract

This study uses the PISA 2022 ICT questionnaire to examine how students’ ICT interaction behaviours in digital environments predict creative thinking across low, medium, and high performance levels (Class 0–2). Using multi-class classification models with Shapley Additive Explanations (SHAP), we identify key ICT-related predictors spanning information retrieval, digital content creation, media evaluation, collaborative communication, and emotional response, with largely nonlinear and interactive effects. Results show that ICT influences creative thinking less through usage frequency than through the intentionality and complexity of ICT engagement and its sociocultural alignment. High-performing students tend to display more functional and strategic ICT behaviours, whereas low-performing students more often show superficial, recreational, or potentially unethical patterns, with emotional regulation, media literacy, and digital ethics emerging as salient factors. These findings clarify performance-specific ICT–creativity profiles and support targeted digital literacy interventions tailored to students at different ability levels.

## 1 Introduction

Creative thinking is widely recognized as a critical skill for education in the 21st century. According to the OECD for International Student Assessment (PISA), creative thinking is defined as the ability to generate, evaluate, and refine ideas, leading to novel and effective solutions, advances in knowledge, or impactful imaginative expressions (Cignetti and Rabella, 2023; Lucas, 2022). This concept emphasizes cognitive processes in everyday contexts, suggesting creativity can be cultivated through practice and education. In educational settings, creativity helps students interpret information innovatively, motivates learning, and is highly valued for future career development. Recent studies indicate that creativity is often a stronger predictor of literacy and numeracy achievements than traditional indicators like Grade Point Average (GPA) (Kapoor et al., 2025), challenging the misconception that creativity conflicts with academic success (Cignetti and Rabella, 2023).

Globally, educational policies increasingly emphasize student creativity. Notably, OECD introduced the largest-ever creativity assessment within PISA in 2022 (Hernández-Ramos and Araya, 2025; Lucas, 2022), evaluating over 140,000 15-year-olds from more than 60 countries. The assessment covers diverse domains, including written and visual expressions, social interactions, and scientific problem-solving, reflecting key cognitive processes like ideation and divergent thinking (Barbot and Kaufman, 2025). The inclusion of creative thinking in PISA highlights global educational attention to cultivating and measuring creativity, providing valuable data for related research (Goecke et al., 2025).

Following the OECD, Information and Communication Technology (ICT) refers to the digital technologies and services used to create, store, manage, communicate, and share information, including hardware, the Internet, software applications, online platforms, and intelligent tutoring systems (Co-operation and Development, 2023). In the digital age, extensive student use of ICT significantly influences their cognitive development and creativity. While purposeful ICT use can enhance creativity through richer imagination and innovative problem-solving (Northcott et al., 2007), research outcomes remain mixed and highly dependent on instructional strategies and individual learning behaviors (Tang et al., 2022). Active engagement with ICT (e.g., creative tasks, digital creation) can foster creativity, whereas passive or excessive ICT consumption may limit reflective thinking and idea generation, potentially hindering creativity (Müller and Montag, 2024). Given these mixed findings, further large-scale research is needed to clarify precisely how ICT behaviors impact student creativity.

Therefore, the present study investigates the following research questions:

RQ1: Which ICT use features significantly predict different levels of students' creative thinking?

RQ2: How do these influential features specifically impact students' creative thinking performances?

Using PISA 2022 data, the present study categorizes creative thinking into low, medium, and high levels, employing ICT-related questionnaire items as predictors and students’ creative thinking levels as the dependent variable. A multi-class classification model based on eXtreme Gradient Boosting (XGBoost) model—many small decision trees that “vote” together— was built, and Shapley Additive Explanations (SHAP) analysis was utilized to interpret the model predictions (Huang et al., 2024). SHAP treats a prediction as the sum of contributions from each variable and reports, for every student, how strongly each variable pushed the model toward or away from a group. This approach quantifies feature contributions to identify key predictors and their mechanisms, providing empirical evidence and practical insights for leveraging ICT to foster student creativity.

## 2 Creative thinking

Creative thinking is generally defined as the capacity to generate, evaluate, and refine ideas to produce novel and effective solutions and expressions in authentic contexts (Northcott et al., 2007; Pont-Niclòs et al., 2024). In the PISA framework, it is assessed via contextualized open-ended tasks and judged along three dimensions—originality, diversity, and appropriateness—to capture both ideation and usefulness. This operationalization provides a comparable basis for international analyses and for linking creative performance with learning processes (Barbot and Kaufman, 2025). Regarding influencing factors, creativity is shaped by knowledge and skills as well as motivation, personality traits, and environmental conditions; openness, curiosity, and intrinsic motivation often stimulate creative behaviors, whereas climates that encourage exploration, iterative revision, and tolerance of failure support risk-taking and innovation. Foundational accounts similarly emphasize the interplay of individual competence and context, including Amabile’s componential theory highlighting domain-relevant skills, creativity-relevant processes, and intrinsic motivation (Amabile et al., 1996), and Csikszentmihalyi’s systems perspective on person–domain–field interactions (Moneta and Csikszentmihalyi, 1996). The role of educators is pivotal: when teachers value and encourage original ideas, students consistently perform better on creative tasks (Hernández-Ramos and Araya, 2025; Lucas and Spencer, 2017; Rosen et al., 2020).

The development of creative thinking positively relates to deeper content understanding, greater learning flexibility (Cignetti and Rabella, 2023), and it aligns with other higher-order skills such as critical thinking and problem solving that together constitute core competencies for future societies (Mumford and McIntosh, 2017; Sur and Ateş, 2022). Learning-analytics studies further indicate that higher creativity levels correlate strongly with superior academic performance, often exceeding predictive validity of traditional academic indicators like GPA or personality traits (Kapoor et al., 2025). Reflecting this significance, education systems increasingly integrate creativity into curriculum standards and competency frameworks, and the dedicated PISA creative thinking assessment operationalizes these priorities via authentic tasks scored on originality, diversity, and appropriateness, offering comparable evidence and practical levers for instructional improvement (Cignetti and Rabella, 2023). At the same time, findings can vary across contexts and measurement approaches, underscoring the need for large-scale, methodologically transparent research to clarify specific pathways and boundary conditions and to inform targeted pedagogical supports (Grey and Morris, 2024; İdil et al., 2024).

## 3 Application of explainable AI in international large-scale assessment

Explainable Artificial Intelligence (XAI) has emerged in educational data science to clarify outputs from complex predictive models. Researchers commonly utilize non-linear modeling methods like random forests, gradient-boosting trees, and deep neural networks in educational assessments to improve prediction accuracy and discover underlying data patterns. However, these models often function as “black boxes,” providing limited transparency regarding their internal decision-making processes. Consequently, XAI methods such as SHAP have been adopted to provide comprehensible interpretations of complex model predictions (Huang et al., 2024)。.

Among the explainable artificial intelligence (XAI) methods currently applied in educational research, SHAP is a widely recognized and extensively used technique (Islam et al., 2021; Wang and Xiao, 2025). Originating from the concept of Shapley values in cooperative game theory, SHAP views model predictions as outcomes resulting from collaborative contributions by individual features. It calculates each feature’s marginal contribution, thus quantifying feature importance and indicating the direction of its effect on predictions (Huang et al., 2024). Positive SHAP values signify a feature’s positive impact, while negative values indicate suppression. Globally, SHAP evaluates feature importance through average absolute contributions, displayed via bar charts or summary plots. Locally, SHAP clearly identifies features driving individual predictions, elucidating the model’s decision mechanisms. Recent work has begun using SHAP with international assessments to reveal which factors complex models rely on and how changes in a variable’s value relate to performance patterns. For instance, Huang et al. (2024) used an XGBoost decision-tree model combined with SHAP to analyze mathematics literacy data from approximately 34,968 junior-high-school students across six East Asian regions participating in PISA 2022. Their analysis successfully identified the most influential predictors among 151 features. Notably, SHAP’s global explanations not only ranked these significant variables but also intuitively showed how variations in feature values influenced students’ mathematics performance. Additionally, SHAP’s local explanations proved particularly valuable for diagnosing individual student outcomes. By generating SHAP local explanation plots separately for high-achieving and lower-performing students, researchers could pinpoint the positive contributing factors (such as effective learning habits and resource support) and negative influences hindering student performance. Such detailed insights provided practical guidance for personalized instruction, allowing educators to strengthen positive factors and address areas needing improvement, thus enhancing targeted educational interventions.

Therefore, SHAP offers notable advantages due to its robust theoretical foundation, broad model applicability (especially effective with tree-based models like XGBoost), and intuitive visualization outputs. By integrating explainable analyses, large-scale educational assessment studies move beyond a simplistic “prediction accuracy” orientation, delving deeper into the educational significance underlying model predictions (Huang et al., 2024).

## 4 Method

### 4.1 Date

The data for this study were drawn from the PISA 2022 database, comprising a sample of 28,342 students from five East Asian economies—Singapore, Hong Kong-China, Macao-China, Chinese Taipei, and Korea. These regions not only consistently perform well in PISA assessments but also lead globally in digital education and the application of educational technologies. Their strong emphasis on ICT integration provides an ideal context for exploring the relationship between students’ ICT interaction and their creative thinking performance. In this study, ICT interaction indicators from the PISA 2022 student background questionnaire—covering Access, Use, and Competencies—were selected as key predictor variables. Students’ creative thinking performance served as the dependent variable. Specifically:

#### 4.1.1 Dependent variable

Students’ creative thinking performance was treated as the target outcome variable, calculated using the average of plausible values (PVs) from the OECD’s official dataset. To enhance the reliability of data-driven analysis and improve interpretability, we adopted the OECD’s six-level classification of creative thinking and regrouped students into three broader levels based on their percentile rankings: high-level group (top 27%), mid-level group (middle 51.3%), and low-level group (bottom 21.7%) (OECD, 2024). Smoothed thresholds were applied to ensure sufficient sample size within each category and meaningful distinctions between groups.

Students in the high-level group were able to generate original, diverse ideas suitable for complex expression and problem-solving tasks. They often produced innovative and abstract outputs, even in unfamiliar contexts. The mid-level group demonstrated the ability to develop appropriate and moderately creative responses, though with limited novelty or abstraction. In contrast, the low-level group primarily produced simple, conventional ideas based on familiar scenarios, reflecting an early stage of creative development. These three groups were labeled as Class 0 (low), Class 1 (medium), and Class 2 (high) respectively. This classification approach enables us to focus on the distinct characteristics of students at each creative level and to explore how ICT interaction mechanisms differ across these groups.

#### 4.1.2 Predictor variables

The study utilized ICT interaction items from the PISA 2022 background questionnaire (items IC170–IC183) as independent variables. These items assessed students’ access to ICT resources, their patterns of ICT use (both in and out of school, for academic and leisure purposes), and their self-reported ICT competencies. ICT interaction represents a dynamic process spanning from access to technology, through usage, to competence development. It serves as a critical link between technology and education, playing a significant role in shaping students’ learning outcomes and innovation potential.

This study investigates whether students’ ICT interaction predicts their creative thinking levels, identifies key influencing features, and explores how these factors shape creative performance.

### 4.2 Data analysis

This study adopts an educational data mining framework consisting of four stages: data preprocessing, feature analysis, model training, and model interpretation. The process integrates logical reasoning and statistical analysis to uncover associations among variables and predict outcomes from large-scale datasets. We first screened associations between ICT features and group labels using chi-square tests, then trained a tree-based model to rank variables by how often they were used to split the data—an intuitive proxy for influence in the learned decision rules. We retained the top features for interpretation and mechanism analysis.

#### 4.2.1 Data preprocessing and label construction

This study is based on the PISA 2022 dataset and focuses on systematically cleaning ICT-related features and constructing classification labels. First, values ranging from 95 to 100—deemed non-informative—were masked and converted into missing values (NaN) to eliminate noise. Samples containing missing values were subsequently removed to ensure data completeness. To construct valid labels, we used the CRT_AVG_10 field and designed a custom classification function. Students were grouped into three levels of creative thinking based on their score distribution: low-performing (bottom 21.7%), medium-performing (middle 51.3%), and high-performing (top 27%). These were labeled as Class 0 (low), Class 1 (medium), and Class 2 (high), respectively, to facilitate multiclass modeling. This classification was informed by the OECD’s official level descriptions to ensure educational interpretability. After multistage filtering, we obtained a high-quality dataset with 17,327 samples and 106 features for subsequent modeling.

#### 4.2.2 Feature selection and correlation analysis

To preliminarily examine the relationship between ICT behavior features and creative thinking performance, chi-square tests were conducted to analyze the statistical significance between categorical variables and class labels. Results showed that all features had *p*-values less than 0.05, indicating significant associations. However, traditional methods fail to capture complex nonlinear or interaction effects. Thus, we employed the XGBoost tree-based model to quantify feature importance. Specifically, we used the “split count” approach, which measures the frequency with which each feature is used as a splitting node in decision trees—providing an intuitive and efficient way to identify key predictors. The top 10 most important features were retained for model interpretation and mechanism analysis.

#### 4.2.3 Model training and performance evaluation

To compare model performance in multiclass prediction tasks, we employed three tree-based machine learning algorithms: Random Forest, LightGBM, and XGBoost. To address class imbalance, we used the Adaptive Synthetic Sampling (ADASYN) method to augment minority classes and enhance model recognition for low-frequency groups. A 10-fold cross-validation strategy was implemented: the dataset was split into 10 subsets, with each fold using nine subsets for training and one for validation, rotating ten times. This ensured both stability and generalizability. After evaluation, XGBoost outperformed the others in accuracy and macro F1-score and was selected as the main classifier. XGBoost training included specific hyperparameters: max_depth = 8, eta = 0.09, n_estimators = 500, with regularization terms alpha = 1 and lambda = 2 to reduce overfitting. The model achieved strong accuracy on the test set. Throughout iterative training and validation, test accuracy from each fold was recorded and averaged. Confusion matrices were constructed to examine classification outcomes across classes, calculating metrics such as false positives, false negatives, precision, recall, and overall performance indicators. To address class imbalance, we used Adaptive Synthetic Sampling (ADASYN), which synthesizes additional examples for under-represented groups so the model learns them better. Performance was compared under internal and external validation. Full model settings (learning rate, depth, regularization) appear in the Supplement; here we focus on accuracy, precision, recall and macro F1.

#### 4.2.4 Model interpretation and visualization

To enhance model transparency and interpretability, we employed the SHAP framework. Using TreeExplainer, we computed the SHAP value for each sample and class, quantifying how each feature contributes to the prediction. We generated SHAP bar plots and beeswarm plots for each class to show the direction and magnitude of feature influence. These visualizations intuitively reveal both positive and negative contributions, as well as potential nonlinear relationships. In addition, scatter plots and marginal density distributions were used to display complex feature–SHAP interactions. Interpretation was conducted at both global and local levels. Globally, SHAP bar charts and summary plots revealed average importance and directional effects across all samples. Locally, individual prediction explanation plots were created for representative samples from each class, illustrating how specific ICT behaviors jointly shaped predictions. SHAP scatter and distribution plots further uncovered nonlinear patterns, revealing that certain features impact creative thinking in complex and layered ways.

In sum, through rigorous data preprocessing, targeted class-imbalance handling, efficient XGBoost training, and systematic SHAP interpretation, we developed a robust and interpretable multiclass prediction framework. This framework significantly enhances the accuracy and transparency of ICT-based classification tasks in the PISA dataset and holds high practical and research value for scalable educational applications. Our XGBoost model is used for predictive classification; SHAP explanations attribute model predictions to features under the observed data distribution. These attributions are not causal effects.

## 5 Results

### 5.1 RQ1: which ICT use features significantly predict different levels of students’ creative thinking?

#### 5.1.1 Model selection results

The performance of three classification models—LightGBM, XGBoost, and Random Forest—on the multi-class classification task is presented in Table 1. To comprehensively evaluate each model’s performance under different datasets and validation methods, the experiments were divided into four groups: (1) training data without ADASYN + internal validation, (2) training data without ADASYN + external validation, (3) training data with ADASYN + internal validation, and (4) training data with ADASYN + external validation. For each group, precision, recall, and F1-score were compared, with the best-performing values in each category highlighted for emphasis. The results show that the XGBoost model consistently outperformed the others across all experimental settings, demonstrating its advantage in handling multi-class classification tasks.

**Table 1:** Performance of LightGBM, XGBoost, and random forest under internal and external validation, with and without ADASYN augmentation.

Validation methods	Model	Accuracy	Low			Middle			High		
			Precision	Recall	F1-score	Precision	Recall	F1-score	Precision	Recall	F1-score
Internal Validation	Lightgbm_noadasyn	0.6837	**0.4724**	0.0545	0.0977	0.6332	**0.7402**	**0.6826**	**0.7458**	0.7115	0.7282
	Xgboost_noadasyn	**0.6839**	0.4213	**0.0757**	**0.1284**	**0.6369**	0.7276	0.6792	0.7427	**0.7211**	**0.7317**
	Randomforest_noadasyn	0.6723	0.4322	0.0609	0.1067	0.6275	0.7137	0.6678	0.7262	0.7122	0.7191
	Support		356			2,644			2,669		
	Lightgbm_adasyn	0.7747	0.9562	0.8985	0.9265	0.6415	0.7000	**0.6829**	0.7393	0.7204	0.7297
	Xgboost_adasyn	**0.7790**	**0.9610**	0.9043	0.9318	0.6469	**0.7025**	0.6735	**0.7412**	**0.7248**	**0.7329**
	Randomforest_adasyn	0.7777	0.9600	**0.9257**	**0.9426**	**0.6486**	0.6937	0.6704	0.7297	0.7078	0.7186
	Support		11,029			10,363			10,897		
External validation:	Lightgbm_noadasyn	0.6768	0.4667	0.0393	0.0725	0.6325	**0.7421**	**0.6829**	**0.7335**	0.6973	0.7149
	Xgboost_noadasyn	**0.6797**	0.4412	**0.0843**	**0.1415**	**0.6413**	0.7209	0.6788	0.7292	0.7182	**0.7237**
	Randomforest_noadasyn	0.6760	**0.5370**	0.0815	0.1415	0.6396	0.7107	0.6732	0.7187	**0.7209**	0.7198
	Support		356			2,644			2,669		
	Lightgbm_adasyn	0.6768	0.4667	0.0393	0.0725	0.6325	0.7421	0.6829	0.7335	0.6973	0.7149
	Xgboost_adasyn	**0.6763**	0.3810	0.1798	0.2443	**0.6477**	**0.6959**	**0.6709**	**0.7256**	**0.7231**	**0.7243**
	Randomforest_adasyn	0.6606	**0.4062**	**0.2191**	**0.2847**	0.6329	0.6800	0.6556	0.7090	0.7003	0.7046
	Support		11,029			10,363			10,897		

The table reports accuracy, recall, and F1-score across four settings: (1) original training data with internal validation, (2) original training data with external validation, (3) ADASYN-augmented data with internal validation, and (4) ADASYN-augmented data with external validation. “Low,” “Middle,” and “High” refer to students with low, medium, and high creative thinking performance, respectively. The number of students in each group varies slightly across conditions due to data preprocessing and sampling adjustments. Bold values indicate the best-performing (highest) value for each metric within each validation method, across the compared models.

Reading Table 1: we compare three models on classifying low/medium/high groups. “Precision” is how often a predicted group is correct; “recall” is how many students in a group the model successfully finds. XGBoost performed best overall, especially after balancing the training data with ADASYN and using external validation.

To examine how students’ ICT interaction influences creative thinking across the “Low,” “Middle,” and “High” groups, it is essential to ensure that the classification models perform well across all categories. As shown in Table 1, the recall and F1-scores for the “Low” group were consistently low under both *internal* and *external* validation when using the original, unbalanced training data. This indicates that the models struggled to correctly identify low-performing students, misclassifying a large proportion of true positives as negatives. Even under the “ADASYN-augmented data + external validation” setting, the LightGBM model demonstrated limited improvement in classifying the “Low” group. Notably, LightGBM performance remained relatively stable regardless of whether the training data was balanced, suggesting that it may be less sensitive to class imbalance. However, the overall classification performance remained affected by the skewed data distribution, particularly for underrepresented categories. In contrast, XGBoost consistently outperformed the other models across all four experimental settings. Its performance was especially strong under the “ADASYN-augmented data + external validation” condition. Based on these results, we selected XGBoost as the primary model for further analysis, using the ADASYN-balanced training data and external validation to ensure robustness and generalizability when identifying key ICT features that influence students’ creative thinking development.

#### 5.1.2 Key ICT interaction features predictive of different levels of creative thinking

**Figure 1:**
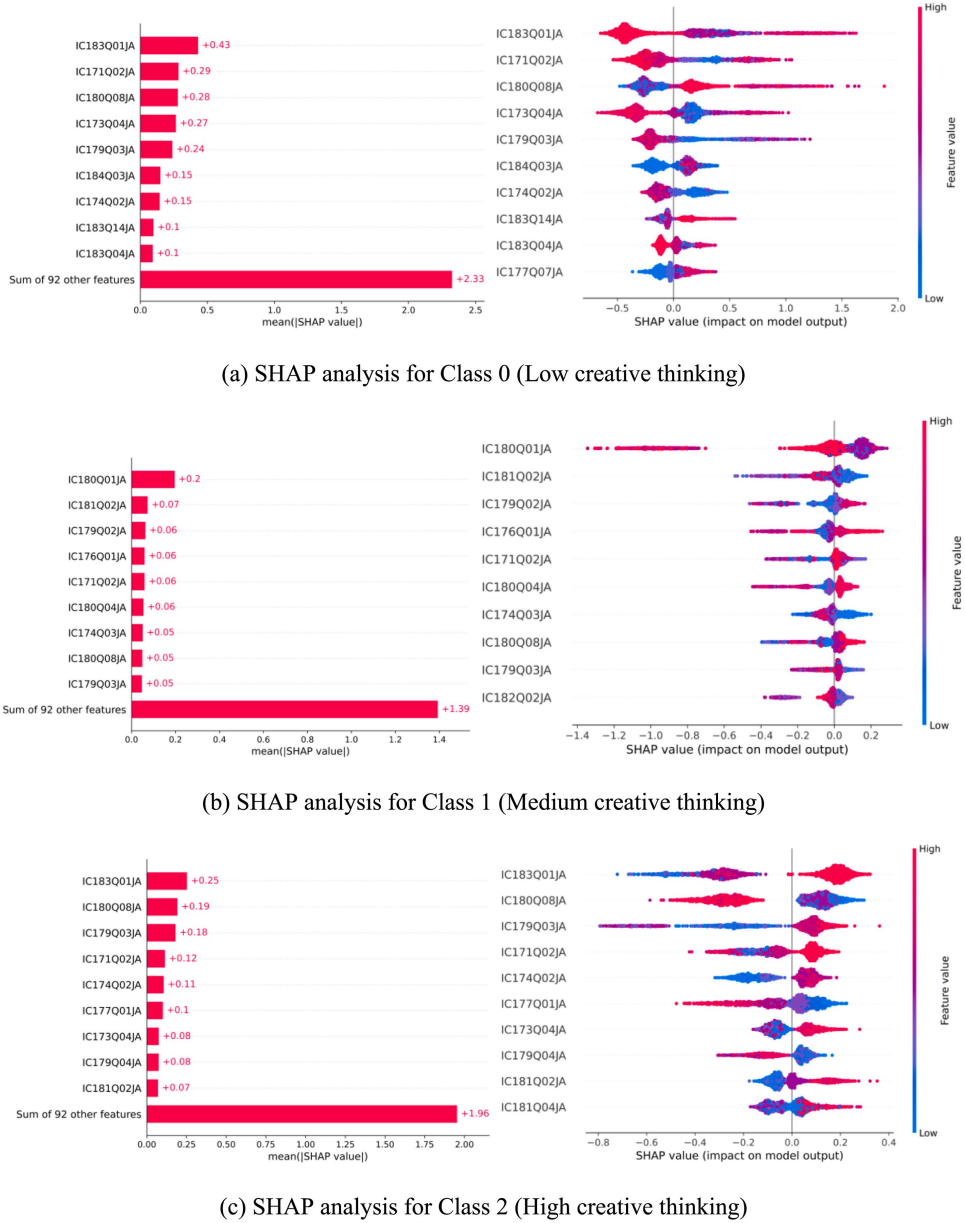
SHAP Analysis of ICT Interaction Features for Predicting Student Creative Thinking Levels. **(a)** SHAP analysis for Class 0 (Low creative thinking). **(b)** SHAP analysis for Class 1 (Medium creative thinking). **(c)** SHAP analysis for Class 2 (High creative thinking). Each point is a student. The horizontal axis shows the survey response; the vertical axis shows how strongly that response pushed the model toward or away from a group (SHAP value). The smooth line summarizes the overall trend; the distribution shape indicates where most students fall.

To identify the most influential ICT interaction features for predicting students’ creative thinking performance, we first used XGBoost to rank feature importance and extracted the top 10 predictors. As shown on the left of Figure 1, the red bars indicate each feature’s relative importance in the model. The right side of Figure 1 presents a SHAP summary plot, which visualizes the average impact of each feature on model predictions across all samples. The Y-axis lists the top 10 features, while the X-axis represents SHAP values, indicating each feature’s direction and magnitude of influence. Each dot corresponds to a sample’s SHAP value for a specific feature. Color reflects the original feature value: red denotes higher values, and blue indicates lower values. The position along the X-axis shows whether a feature contributes positively or negatively to the prediction. Violin-shaped patterns illustrate the distribution and density of SHAP values for each feature, revealing their variability and consistency across the dataset.

Through SHAP analysis, we extracted the contribution of each feature to the model’s classification of the three creative thinking levels (low, medium, and high). Overall, certain features consistently demonstrated high importance across all categories, serving as key factors in the model’s multiclass discrimination. For example, two features —IC179Q03JA (co-developing device-use rules (attitude)) and IC180Q08JA (sharing made-up information without flagging inaccuracy) ranked among the top in terms of mean absolute SHAP values across all classes, indicating their substantial influence in predicting any creative thinking level. This suggests that these ICT-related behaviors are closely associated with students’ creative performance and play a central role in distinguishing between groups. In Class 0 (low creativity), features IC177Q07JA (time spent on digital creation (weekday)) and IC183Q04JA (collaboration self-efficacy) significantly increased the model’s likelihood of assigning this label (Figure 1a). In Class 1 (medium), high values of IC182Q02JA (interest in learning programming) and IC180Q04JA (discussing the accuracy of online information in class) strongly pushed predictions toward this category (Figure 1b). For Class 2 (high), IC181Q04JA (emotional response to online privacy violations) and IC179Q04JA (support for restrictive device policies) showed notable positive contributions to the model’s outputs (Figure 1c). Some features, such as IC171Q02JA (smartphone use outside school) and IC183Q01JA (online information-search self-efficacy), exhibited moderate importance across categories, suggesting a generalizable support role within the model structure. Although not the most dominant predictors, they provided stable informational value, enhancing the model’s discriminative capacity across all levels. Overall, the same feature may exert different effects—both in direction and magnitude—depending on the predicted class, reflecting the model’s nuanced response to the distinct patterns among the groups.

Table 2 presents the key variable codes and corresponding questionnaire items.

**Table 2:** Key ICT interaction features predictive of different levels of creative thinking.

Class	Variable code	Content
0	IC183Q01JA	To what extent are you able to search for and find relevant information online?
	IC171Q02JA	This school year, how often did you use a smartphone (i.e., mobile phone with Internet access) outside of school?
	IC180Q08JA	To what extent do you agree: I share made-up information on social networks without flagging its inaccuracy?
	IC173Q04JA	How often do you use digital resources in computer science / information technology / informatics lessons?
	IC179Q03JA	To what extent do you agree: Students should collaborate with teachers to decide on the rules regarding digital device use during lessons?
	IC184Q03JA	How often do you use digital resources for simulations and modelling (e.g., GeoGebra, NetLogo), virtual laboratories (e.g., Labster) in mathematics?
	IC174Q02JA	How often did you use digital resources to write or edit text for a school assignment (e.g., using Google Docs, MS Word)?
	IC183Q14JA	To what extent are you able to create a computer program (e.g., in Scratch, Python, Java)?
	IC183Q04JA	To what extent are you able to collaborate with other students on a group assignment?
	IC177Q07JA	During a typical weekday, how much time do you spend to create or edit your own digital content (pictures, videos, music, computer programs)?
1	IC180Q01JA	To what extent do you agree: I trust what I read online.
	IC181Q02JA	How upset were you the last time this happened: Encountering discriminatory content online (e.g., about race, gender, sexual orientation, or appearance)?
	IC179Q02JA	To what extent do you agree: Students should not be allowed to bring their own laptop (or tablet) to class.
	IC176Q01JA	This school year, how often did you use digital resources to see your grades or results from assignments (e.g., homework/tests)?
	IC171Q02JA	This school year, how often did you use a smartphone with Internet access outside of school?
	IC180Q04JA	To what extent do you agree: I discuss the accuracy of online information with my teachers or in class.
	IC174Q03JA	How often did you use digital resources to find information online about real-world problems or phenomena (e.g., climate change, oil spills)?
	IC180Q08JA	To what extent do you agree: I share made-up information on social networks without flagging its inaccuracy.
	IC179Q03JA	To what extent do you agree: Students should collaborate with teachers to decide on rules regarding digital device use during lessons.
	IC182Q02JA	To what extent do you agree: I am interested in learning computer programming.
2	IC183Q01JA	To what extent are you able to search for and find relevant information online?
	IC180Q08JA	To what extent do you agree: I share made-up information on social networks without flagging its inaccuracy?
	IC179Q03JA	To what extent do you agree: Students should collaborate with teachers to decide on rules for digital device use during lessons?
	IC171Q02JA	This school year, how often did you use a smartphone (with Internet access) outside of school?
	IC174Q02JA	How often did you use digital resources to write or edit text for a school assignment (e.g., Google Docs, Word)?
	IC177Q01JA	During a typical weekday, how much time do you spend playing video games (on smartphone, console, etc.)?
	IC173Q04JA	How often do you use digital resources in computer science / information technology / informatics lessons?
	IC179Q04JA	To what extent do you agree: The school should set up filters to prevent students from going on social media?
	IC181Q02JA	How upset were you the last time you encountered discriminatory content online?
	IC181Q04JA	How upset were you the last time information about you was publicly displayed online without your consent?

**Figure 2:**
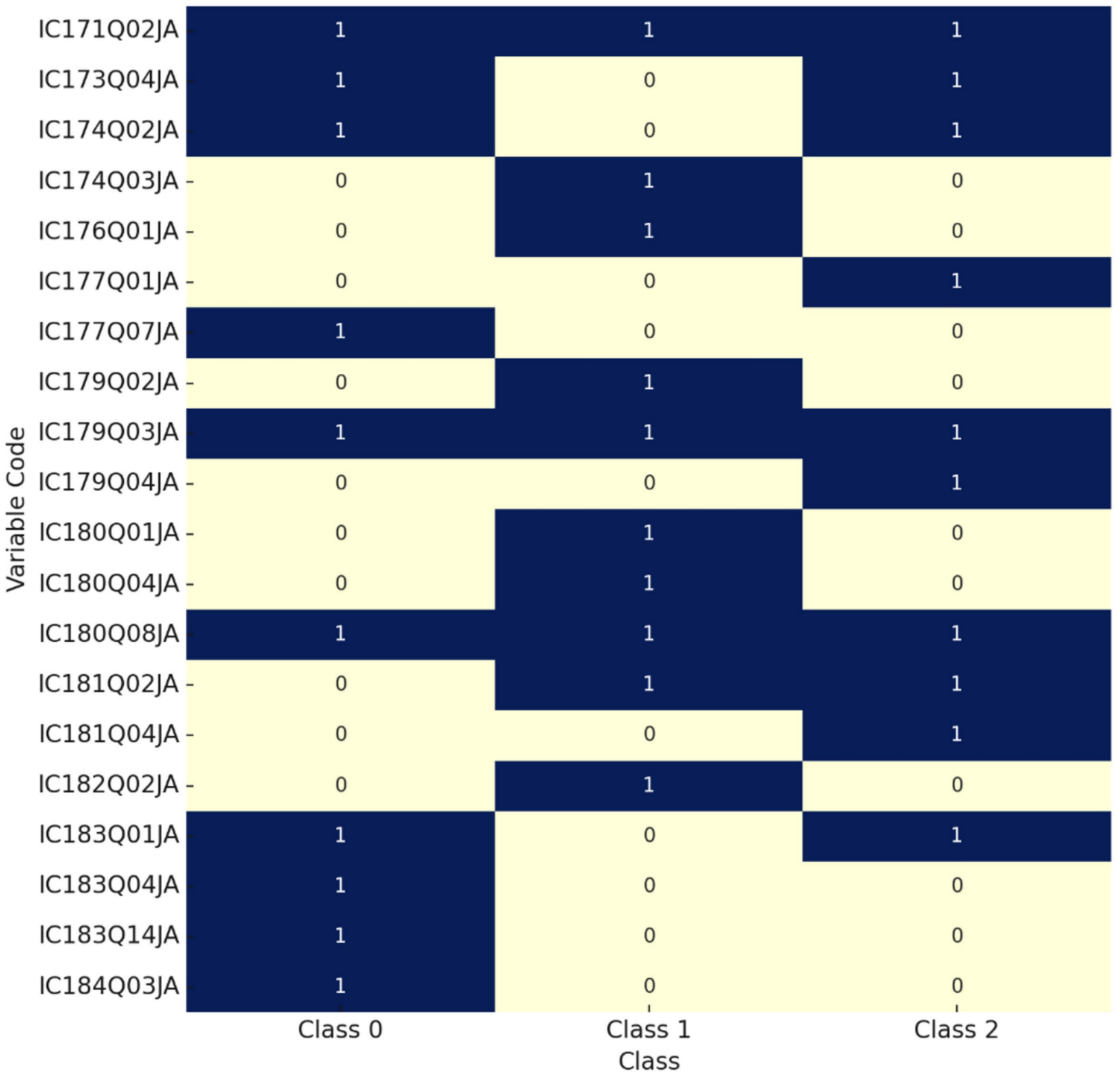
Presence of variables across classes.

Figure 2 illustrates the presence of key ICT-related variables across three student groups with differing levels of creative thinking (Class 0, 1, and 2). Each row represents an ICT interaction variable (indicated by its questionnaire code), and each column corresponds to a student class. Dark blue cells (value = 1) indicate that the variable was identified among the top 10 most important features for that class, while light yellow cells (value = 0) denote non-selection. As shown in the figure, there is both overlap and divergence in variable importance across the three groups. For instance, IC171Q02JA (frequency of smartphone use outside of school) appears in all three classes, suggesting its broad predictive value regardless of students’ creativity level. In contrast, IC183Q14JA (ability to write computer programs) and IC183Q04JA (collaboration on group tasks) are uniquely important for Class 0, indicating that these digital skills may be particularly relevant for distinguishing students with lower levels of creative thinking. Moreover, some features are shared between Class 1 and Class 2, such as IC180Q08JA (sharing made-up information online) and IC181Q02JA (exposure to discriminatory content online), implying behavioral similarities in information judgment and digital experiences among students with moderate to high creativity. On the other hand, variables exclusive to Class 1—such as IC182Q02JA (interest in learning programming)—may reflect group-specific characteristics in cognitive motivation.

### 5.2 RQ2: how do these influential features specifically impact students’ creative thinking performances?

Based on the SHAP analysis results for each creative thinking level, we describe the key feature impact patterns and predictive contributions corresponding to the low-level creativity group (Class 0), the medium-level group (Class 1), and the high-level group (Class 2), respectively.

**Figure 3:**
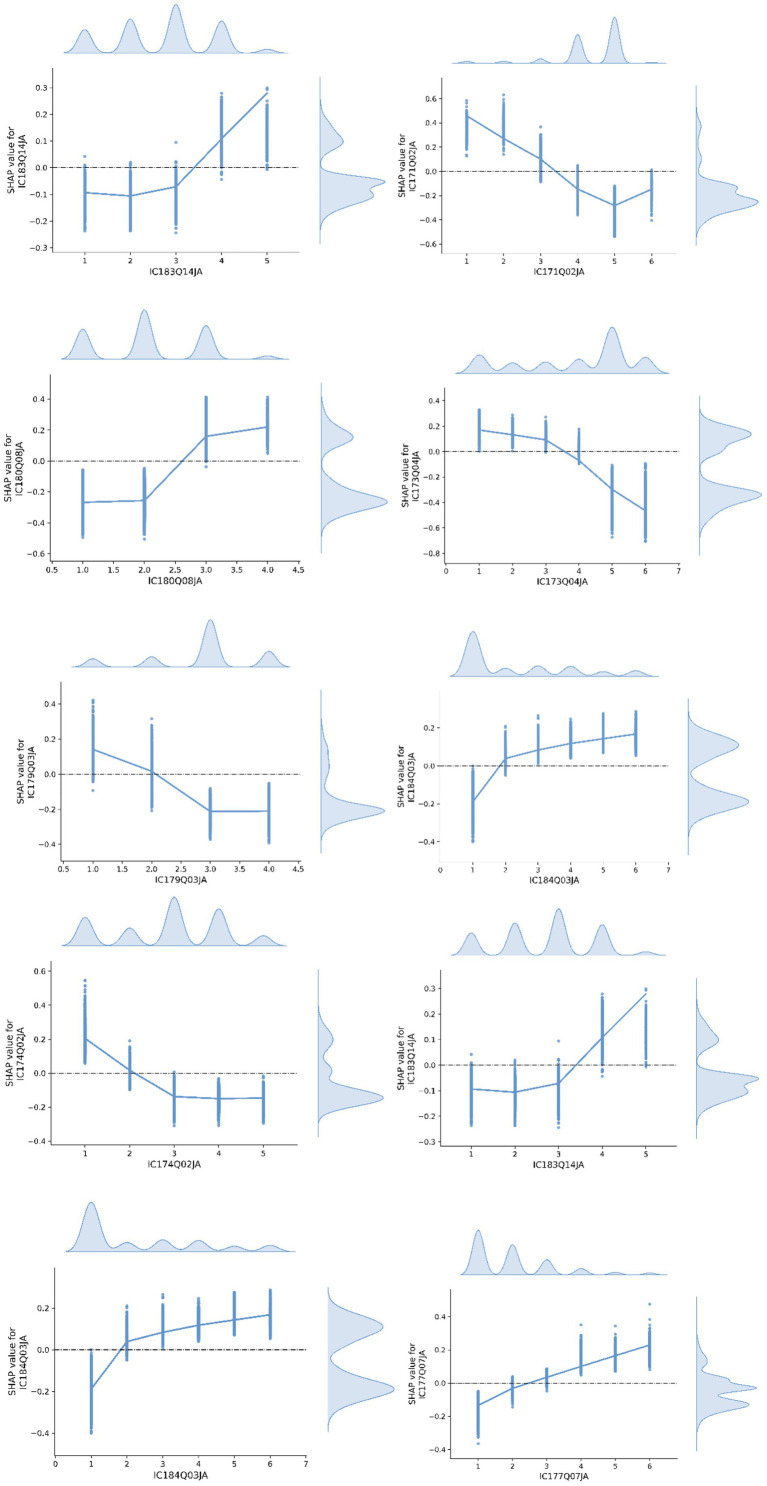
SHAP value relationship plot of features in Class 0.

Figure 3 illustrates the SHAP value relationship plots for the most influential features identified in Class 0. The following paragraphs provide detailed interpretations of each feature’s contribution and its trend characteristics.

IC183Q01JA (Online information-search self-efficacy): This item assesses students’ ability to search for and locate relevant information online. The response options range from 1 (“Not at all”) to 4 (“Easily”), with 5 indicating “I do not know what this is.” SHAP analysis revealed a clear upward trend in SHAP values as response scores increased from 1 to 4, indicating a positive relationship. This suggests that stronger information retrieval skills are more predictive of students being classified into Class 0 (low creative thinking group), highlighting the prevalence of this basic digital competency within this subgroup.

IC171Q02JA (Smartphone use outside school): This item measures how often students used an internet-enabled smartphone outside of school during the academic year, with response options ranging from 1 (“Never”) to 6 (“No access to the device”). The SHAP results show that lower values—indicating less frequent use—correspond to higher SHAP values. Thus, students who rarely or never used smartphones were more likely to be classified into Class 0. This finding objectively reflects a potential association between digital access and creative thinking, particularly that limited exposure to digital environments may constrain creativity development.

IC180Q08JA (Sharing made-up information without flagging inaccuracy): This item asks students to what extent they agree with the statement “I share made-up information on social networks without flagging its inaccuracy,” with response options ranging from 1 (“Strongly disagree”) to 4 (“Strongly agree”). SHAP values increase linearly with the level of agreement, indicating that students who are more likely to engage in this behavior are more often classified into Class 0. The result suggests that a lack of media literacy or responsibility in digital communication may be a salient characteristic of low-creativity students.

IC173Q04JA (Use of digital resources in CS/IT class): This item assesses how often students use digital resources in computer science or information technology classes, with options ranging from 1 (“Almost never”) to 5 (“In almost every lesson”), and 6 for “Did not take this subject.” The SHAP values decrease as the frequency increases, indicating that frequent digital engagement in formal coursework is negatively associated with Class 0 classification. This implies that active digital participation in structured learning contexts may serve as a differentiating factor for higher creative engagement.

IC179Q03JA (Co-developing device-use rules, attitude): This item gauges whether students agree that rules regarding digital device use during lessons should be co-developed by students and teachers, with options ranging from 1 (“Strongly disagree”) to 4 (“Strongly agree”). The SHAP values display a U-shaped distribution, with higher predictive power at both ends of the scale. This indicates that students holding polarized views—either strongly agreeing or strongly disagreeing—show more distinct classification patterns, and may reflect greater variability in classroom norm perceptions within Class 0.

IC184Q03JA (Use of simulations/modeling in mathematics): This item asks how often students use digital tools for simulations or modeling in mathematics (e.g., GeoGebra, virtual labs), with responses from 1 (“Never”) to 5 (“Every day”), and 6 (“Not applicable”). SHAP values rise with increasing frequency of use, suggesting that students who frequently engage in advanced digital learning tools are less likely to be classified as Class 0. The result points to a potential relationship between digital learning complexity and creative thinking differentiation.

IC174Q02JA (Digital writing frequency, Word/Docs): This item captures the frequency with which students use digital tools (e.g., Google Docs, MS Word) to write or edit text for school assignments. Options range from 1 (“Almost never”) to 5 (“Almost every day”). SHAP values are higher at lower response levels, indicating that infrequent users are more likely to fall into Class 0. This highlights the relevance of digital expression and written communication as indicators of creative engagement, with limited usage signaling lower levels of creative output.

IC183Q14JA (Programming self-efficacy): This item assesses students’ self-reported ability to write computer programs using tools such as Scratch, Python, or Java, with response options from 1 (“Not at all”) to 4 (“Easily”), and 5 (“Do not know what this is”). A significant jump in SHAP values is observed at option 4, with minimal variation for other values. This suggests that students with clear programming proficiency are less likely to belong to Class 0, and this variable holds distinct predictive power in distinguishing lower- from higher-performing groups.

IC183Q04JA (Collaboration self-efficacy): This item asks to what extent students can collaborate with peers on group assignments, with options from 1 (“Not at all”) to 4 (“Easily”), and 5 (“Do not know what this means”). SHAP values increase with higher self-rated collaboration ability. This positive association indicates that students with stronger social and cooperative learning skills are less likely to belong to the low creativity group, reinforcing the role of collaborative competence in creative development.

IC177Q07JA (Time spent on digital creation, weekday): This item measures how much time students spend on a typical weekday creating or editing their own digital content (e.g., pictures, videos, music, programs), with response options from 1 (“None”) to 6 (“More than 7 h”). The SHAP analysis shows a clear positive trend, where increased time spent is associated with lower likelihood of being in Class 0. This suggests that frequent engagement in self-initiated digital creation may be an important behavioral marker of creative thinking potential.

Overall, students in the low-creativity group tend to show limited or lower-quality ICT engagement—such as infrequent use of digital writing tools and higher tolerance for sharing inaccurate information—whereas productive practices like programming proficiency, collaborative work, simulations/modeling, and self-initiated content creation consistently differentiate students away from this group. A notable nuance is that basic information-search self-efficacy appears relatively common here, suggesting that foundational skills alone are insufficient for creative development unless coupled with purpose-driven, higher-quality ICT activities.

**Figure 4:**
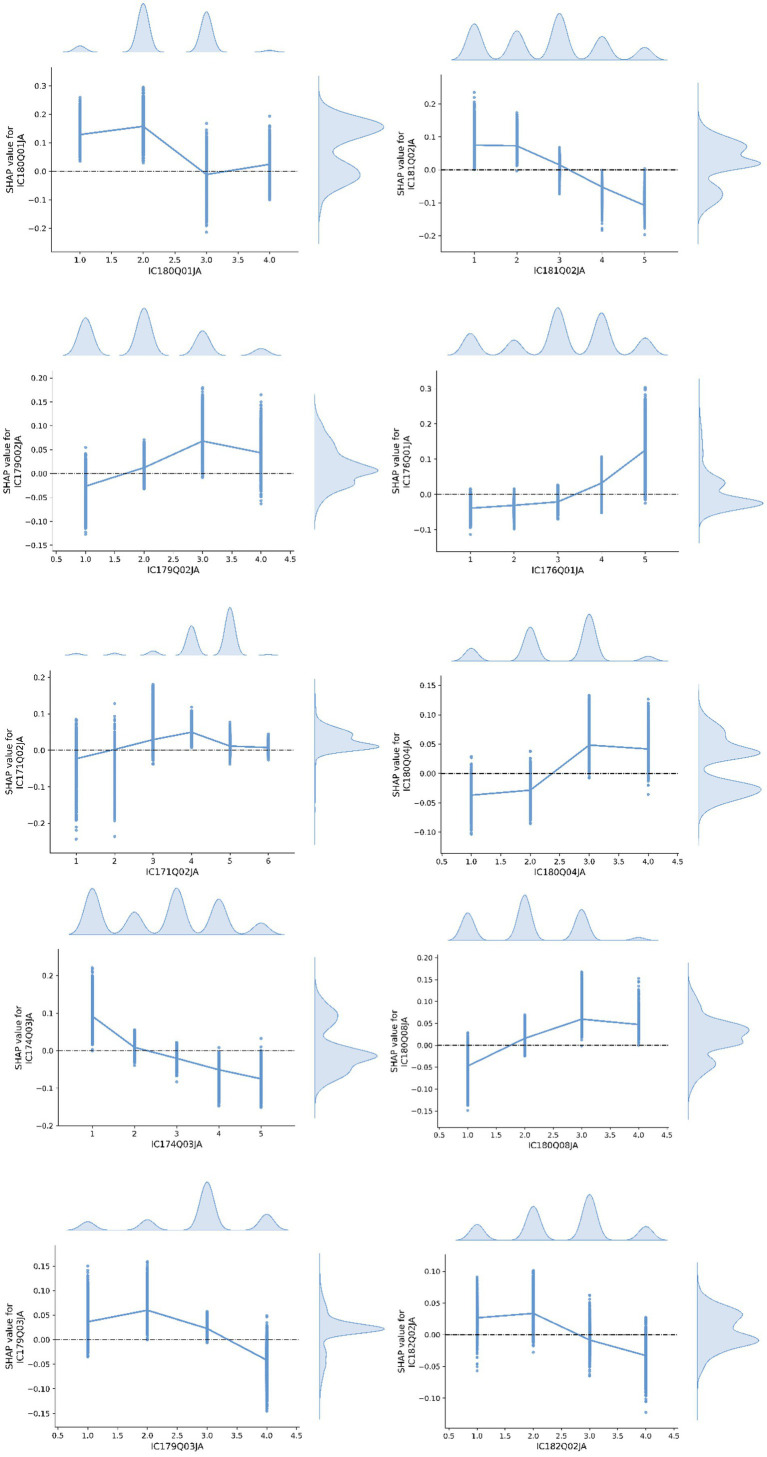
SHAP value relationship plot of features in Class 1.

Figure 4 illustrates the SHAP value relationship plots for the most influential features identified in Class 1. The following paragraphs provide detailed interpretations of each feature’s contribution and its trend characteristics.

IC180Q01JA (Trust in online information): This item assesses students’ agreement with the statement, *“I trust what I read online,”* on a scale from 1 (strongly disagree) to 4 (strongly agree). The SHAP analysis reveals a non-linear pattern, with slightly higher SHAP values at intermediate response levels (2–3), and smaller impacts at the extremes. This suggests that students who hold moderate or cautiously trusting attitudes toward online information are more likely to be classified into Class 1, potentially reflecting a balanced and reflective approach to digital content evaluation.

IC181Q02JA (Emotional distress at discriminatory online content): This item measures the emotional distress students felt when last encountering discriminatory content online (e.g., related to race, gender, appearance), with options ranging from 1 (not at all upset) to 5 (very upset). The SHAP values decrease consistently with higher response levels, indicating that students with stronger emotional reactions are less likely to be classified as Class 1. This may reflect a relatively lower level of emotional sensitivity or a more detached response style among students in the medium creativity group.

IC179Q02JA (Support for banning BYOD in class): This item examines students’ attitudes toward the statement, *“Students should not be allowed to bring their own laptops or tablets to class,”* on a scale of 1 (strongly disagree) to 4 (strongly agree). The SHAP distribution exhibits an inverted U-shaped curve, with the highest contribution to model prediction at moderate agreement levels (responses 2–3). This pattern suggests that students with balanced views on device policy are more representative of the Class 1 group.

IC176Q01JA (Checking grades/assignment results with digital tools): This item explores how often students used digital tools during the school year to check grades or assignment results, with options ranging from 1 (almost never) to 5 (almost every time). SHAP values increase linearly with frequency, indicating that students who more frequently engage with digital performance feedback are more likely to be predicted as Class 1. This highlights the role of self-monitoring through digital resources as a relevant behavioral feature.

IC171Q02JA (Smartphone use outside school): This item measures how often students used an internet-enabled smartphone outside of school, with options from 1 (almost never) to 5 (daily), and 6 indicating lack of access. The SHAP trend peaks around mid-range responses (3–4) and drops significantly at both ends. This suggests that students with moderate smartphone usage are more likely to fall within the medium creativity group, potentially reflecting a balanced level of digital exposure.

IC180Q04JA (Discussing the accuracy of online information in class): This item evaluates students’ agreement with the statement, *“I discuss the accuracy of online information with my teachers or in class,”* rated from 1 (strongly disagree) to 4 (strongly agree). SHAP values increase with agreement, with a notable rise at level 4. This finding implies that students actively engaged in critical discussions about information credibility are more likely to belong to Class 1.

IC174Q03JA (Using digital resources to research real-world issues): This item assesses how frequently students used digital resources to research real-world issues (e.g., climate change, oil spills), with options from 1 (almost never) to 5 (almost every day). The SHAP values decrease with increasing frequency, showing a negative correlation. Students who engage in such research activities more frequently are less likely to be classified into Class 1, which may suggest lower engagement with applied digital inquiry tasks among this group.

IC180Q08JA (Sharing made-up information without flagging inaccuracy): This item measures students’ agreement with the statement, *“I share made-up information on social networks without flagging its inaccuracy,”* on a scale from 1 (strongly disagree) to 4 (strongly agree). SHAP values rise with increased agreement, suggesting that students who admit to sharing inaccurate content are more likely to be predicted as belonging to Class 1. This may reflect varying degrees of media literacy or awareness within this group.

IC179Q03JA (Co-developing device-use rules, attitude): This item evaluates students’ agreement with the statement, *“Students should collaborate with teachers to decide on rules regarding digital device use during lessons,”* with response options from 1 (strongly disagree) to 4 (strongly agree). SHAP values peak at intermediate responses (2–3), reflecting a preference for cooperative rule-making among moderately creative students.

IC182Q02JA (Interest in learning programming): This item asks whether students are interested in learning computer programming, rated from 1 (strongly disagree) to 4 (strongly agree). SHAP values show a clear upward trend with increasing agreement, suggesting that students with a stronger interest in programming are more likely to be assigned to Class 1. This highlights programming interest as a motivational factor in this student group.

The medium-creativity group presents a balanced profile, combining moderate trust in online information and mid-range device use with stronger engagement in digital self-monitoring and classroom discussions of information credibility; however, applied digital inquiry (e.g., researching real-world issues) is less typical, and some tolerance for misinformation remains. Taken together, these patterns point to a transitional stage in which targeted support for inquiry-oriented creation and collaborative production may help shift engagement toward more constructive, goal-directed uses of ICT.

**Figure 5:**
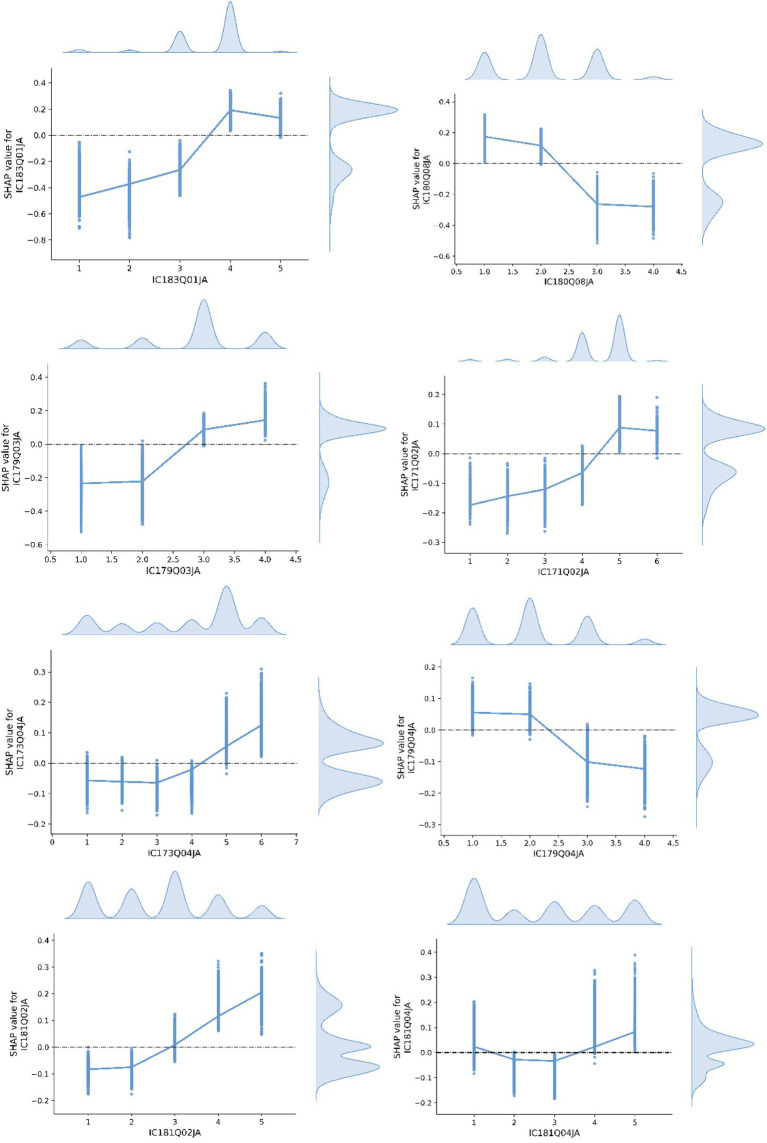
SHAP value relationship plot of features in Class 2.

Figure 5 illustrates the SHAP value relationship plots for the most influential features identified in Class 2. The following paragraphs provide detailed interpretations of each feature’s contribution and its trend characteristics.

IC183Q01JA (Online information-search self-efficacy): Response options range from 1 (Not at all) to 4 (Very easily), with 5 indicating “Do not know what this is.” The SHAP value analysis reveals that this variable shows the strongest discriminative power for Class 2 (high creativity group). The SHAP values increase significantly with higher response options, indicating a clear positive trend. This suggests that students with stronger online information-searching skills are more likely to be classified as highly creative, underscoring the importance of information literacy in cognitive innovation.

IC180Q08JA (Sharing made-up information without flagging inaccuracy): Responses range from 1 (Strongly disagree) to 4 (Strongly agree). The SHAP analysis shows a steady negative trend: higher agreement levels (i.e., more tolerance toward spreading misinformation) are associated with lower SHAP values. This implies that students who condone the dissemination of false information are less likely to be classified as highly creative, suggesting a potential link between creativity and a stronger sense of digital responsibility and ethical awareness.

IC179Q03JA (Co-developing device-use rules, attitude): Responses range from 1 (Strongly disagree) to 4 (Strongly agree). The SHAP values increase with higher levels of agreement, showing a clear positive relationship. This indicates that students who hold more collaborative attitudes are more likely to be predicted as highly creative. Such a pattern may reflect the association between open-mindedness, co-regulation, and creative cognition.

IC171Q02JA (Smartphone use outside school): Response options range from 1 (Never) to 6 (No access). SHAP values generally increase with the response level, peaking at the highest categories. This trend suggests that students with more frequent access to digital devices outside of school are more likely to be classified as creative, possibly due to greater exposure to diverse digital content and tools.

IC174Q02JA (Digital writing frequency, Word/Docs): Response options range from 1 (Almost never) to 5 (Almost every day). The SHAP values show a clear and steady upward trend. This indicates that frequent engagement with digital text composition tools (e.g., Word, Google Docs) is positively associated with creative group classification, highlighting the role of digital written expression in fostering creativity.

IC177Q01JA (Time spent on digital gaming): Responses range from 1 (Never) to 6 (More than 7 h). The SHAP values demonstrate a clear negative trend: students who report longer durations of gaming are less likely to be classified as highly creative. This suggests that excessive time spent on digital entertainment may detract from cognitively enriching or creative activities.

IC173Q04JA (Use of digital resources in CS/IT class): Responses range from 1 (Almost never) to 5 (Almost every lesson), with 6 indicating “Did not take this subject.” The SHAP values increase across response levels, especially at higher frequencies of use. This suggests that consistent use of digital resources in computing-related lessons may enhance the technological fluency associated with creative thinking.

IC179Q04JA (Support for restrictive device policies): Responses range from 1 (Strongly disagree) to 4 (Strongly agree). SHAP values decrease as agreement levels rise, reflecting a negative trend. This indicates that students who support restrictive digital policies are less likely to be classified in the creative group, highlighting a potential association between digital openness and creative expression.

IC181Q02JA (Emotional distress at discriminatory online content): Responses range from 1 (Not at all) to 5 (Very). SHAP values show a consistent positive correlation with the intensity of emotional reaction. This may suggest that students in the high creativity group tend to have stronger social–emotional awareness and empathy when exposed to online injustices.

IC181Q04JA (Emotional response to online privacy violations): Response options are similar to the previous item. SHAP values display a U-shaped pattern, being slightly higher at both ends of the response scale. This non-linear effect suggests that both low and high emotional sensitivity to online privacy violations are somewhat predictive of creative group membership, possibly indicating diverse cognitive-emotional profiles within this group.

The high-creativity group is characterized by strong information literacy and digital responsibility, frequent use of academically productive tools (e.g., digital writing and computing resources), collaborative stances toward classroom rules, and reduced time in entertainment-only activities. Greater out-of-school access and clearer socio-emotional responsiveness further distinguish this group, aligning with a purpose-driven engagement profile that coheres with higher creative performance.

## 6 Discussion

This study utilized data from the PISA 2022 ICT questionnaire to develop an interpretable multi-class classification model and employed SHAP to probe the mechanisms underlying the prediction of students’ creative thinking levels. Through visualizing SHAP values and analyzing nonlinear patterns of ICT-related behavioral variables across three creative thinking groups (Classes 0–2), we identified a set of key predictive features that span multiple dimensions, including information retrieval, digital content creation, collaborative communication, media literacy, and emotional regulation. These variables not only demonstrated strong discriminative power across the three creativity groups but also revealed complex nonlinear and interaction effects. Our findings suggest that the impact of ICT behaviors on creative capacity is not simply linear or frequency-dependent but instead highly contingent on the intentionality, complexity, and contextual relevance of digital practices. Based on these insights, the following discussion draws on existing literature to further examine the functional mechanisms of ICT behaviors in creative development, behavioral divergences across creativity groups, and the roles of emotional regulation, media literacy, and digital ethics.

Practically, our findings indicate that creativity is best supported when ICT use is explicitly goal-aligned and production-oriented; brief guided search with credibility checks, short create–feedback–revise cycles, and structured collaboration convert screen time into visible creative products.

### 6.1 Mechanisms of creativity development in digital contexts

A growing body of research has emphasized the importance of collaboration, communication, and social interaction in the development of creativity from a sociocultural perspective (Selfa-Sastre et al., 2022). Digital technology serves as both a medium and a cultural tool for learning, providing enriched platforms for shared creative engagement. On the one hand, digital tools can act as “collaborative mentors” guiding students in co-creative practices; on the other, they help construct a “cultural space” that enables interactive creativity among groups (Vilarinho-Pereira et al., 2021). Our study found that students in the high-creativity group demonstrated more frequent task-oriented digital behaviors, such as active information seeking, creative expression, and metacognitive engagement—findings that are consistent with prior evidence suggesting that high-constructive use of ICT facilitates creative performance (Tang et al., 2022).

### 6.2 Frequency versus quality of ICT use: toward a functionalist understanding

A growing body of work indicates that frequency of ICT use, by itself, is a poor predictor of creativity; what is more consequential is the quality and goal-directedness of use (Juuti et al., 2022). Extending this view, studies show that technology promotes learning and creative outcomes chiefly when it functions as an effective tool for interactive, learning-aligned goals—for example, inquiry, design, and collaborative production—whereas recreational or superficial usage yields limited benefits and can even be detrimental (Abedini, 2020). This pattern aligns with established theories: under the ICAP framework, interactive/constructive digital activities elicit generative processing and knowledge reorganization to a much greater extent than passive or merely active use (Chi and Wylie, 2014); self-determination theory predicts that goal-aligned tasks that support autonomy, competence, and relatedness heighten intrinsic motivation and persistence (Deci and Ryan, 2000); and self-regulated learning accounts emphasize goal setting, monitoring, feedback, and reflection as mechanisms that transform initial ideation into refined creative products (Zimmerman, 2000). Consistent with these accounts, our results indicate that frequency without clear creative intent or contextual relevance fails to foster creativity, whereas purpose-driven practices—such as inquiry-based exploration, original content generation, and collaborative problem-solving—are reliably associated with stronger creative performance (Juuti et al., 2022). Taken together, these mechanisms suggest that quality often mediates the effect of frequency, and that benefits may exhibit non-linear patterns (e.g., thresholds or inverted-U relationships) contingent on task authenticity, teacher scaffolding, collaboration quality, time allocation, and learners’ prior skills and resources. Applied to profiles, students resembling the low-creativity group benefit from routine digital writing and scaffolded simulations; those in the medium group advance with applied inquiry and regular discussions of information accuracy plus self-monitoring; highly creative students gain most from authentic-audience production and opportunities to mentor peers in collaborative creation.

### 6.3 Behavioral pattern divergence across creativity levels

Students with varying levels of creative thinking exhibited distinct behavioral profiles in digital environments. Highly creative students tended to engage digital tools more proactively and strategically to expand cognitive boundaries, whereas their less creative counterparts often defaulted to passive information consumption. Several studies using objective data and machine learning methods have revealed these differences. Muldner and Burleson used sensor tracking and machine learning to model students’ creative processes during digital geometry proof tasks. Their findings indicated reliable differences in behavioral and physiological characteristics between high- and low-creativity students: those in the high-creativity group exhibited eye movement and physiological patterns associated with sustained focus and cognitive flexibility, which enabled machine classifiers to distinguish between groups with high accuracy (Muldner and Burleson, 2015). Another study by Müller and Montag (2024) examined the impact of smartphone and social media usage on creativity. Their survey of 509 participants revealed that students with higher self-efficacy in creativity tended to use smartphones more moderately and were less prone to overuse messaging apps and platforms like Facebook or Instagram. However, no universal negative correlation was observed between high social media use and performance on divergent thinking tasks; some subgroups even showed positive associations between specific digital behaviors and creativity outcomes. These patterns are consistent with foundational accounts in which creative performance depends on domain-relevant skills, creativity-relevant processes, and intrinsic motivation (Amabile et al., 1996) and emerges from interactions among the person, domain, and field—interactions that digital tools and social platforms can reconfigure (Moneta and Csikszentmihalyi, 1996). Broadly, students with higher creativity levels appear more adept at using productive tools (e.g., computers) for idea generation, while those with lower creativity may be more vulnerable to distraction by fragmented content and exhibit less purposeful behavior. Although findings vary across contexts, there is a consistent argument for considering the bidirectional relationship between creativity and digital behavior. Tailored digital literacy training and usage guidance for different creativity groups may help all students participate in digital interactions more effectively and creatively (Müller and Montag, 2024). In practical terms, classrooms can surface the ethical dimension of creative ICT use through light-touch routines that emphasize quality over frequency: brief credibility checks embedded in inquiry/creation tasks (lateral reading, cross-verification, and noting uncertainty) and explicit attribution/licensing for digital assets. Such routines align with media-literacy principles and have been shown to improve students’ online source evaluation and responsible reuse (Hobbs, 2010). A compact rubric that combines originality–diversity–appropriateness with integrity criteria (accuracy, attribution, privacy) supports formative feedback without inflating screen time.

### 6.4 The role of emotional regulation in ICT–creativity linkages

Beyond behavioral dimensions, students’ emotional and psychological characteristics profoundly shape the effectiveness of ICT use on creativity. Emotional regulation, in particular, has been recognized as a crucial psychological resource in creative processes. Digital creation often involves trial, error, and frustration; students who can regulate their emotions are more likely to persist and draw insights from failure. Studies from the emotional intelligence perspective support this notion. For example, Demirkol et al. found that teachers with higher emotional intelligence—defined as the ability to perceive and manage emotions—were more inclined to employ ICT for creative instruction, with corresponding increases in student creativity. Structural equation modeling showed a positive relationship between emotional intelligence and teachers’ use of the Internet for creativity enhancement, while “computer anxiety” had the opposite effect. This view aligns with the process model of emotion regulation, which posits that antecedent- and response-focused strategies modulate attention and cognitive resources recruited for creative problem solving (Gross, 2015), and with broaden-and-build theory, which proposes that positive emotions expand momentary thought–action repertoires and, over time, build resources relevant to creative work (Fredrickson, 2001). For students, strong emotion regulation skills may enable them to transform ICT into a catalyst for creativity, whereas poor emotional coping may reduce ICT to superficial engagement or even distraction.

### 6.5 Media literacy as a key enabler of creative digital expression

Media literacy, encompassing the ability to access, analyze, evaluate, and create digital content, serves as another critical moderator of ICT’s creative affordances (Al-Zou’bi, 2025). Our findings show that variables related to media judgment and collaborative content production—such as “discussing information accuracy with teachers” or “using platforms for content creation”—were strong predictors of high creativity levels. Al-Zou’bi’s quasi-experimental study demonstrated that university students who received media and information literacy (MIL) training significantly outperformed their peers in digital video creativity tasks. Similarly, Xu et al. (2025) found that art and design students with higher media literacy were more likely to produce original works and achieve superior academic outcomes. These results underscore the importance of cultivating students’ abilities in media discernment and digital expression as a pathway to realizing ICT’s creative potential (Xu et al., 2025). Moreover, digital ethics awareness and online civic literacy serve as essential normative safeguards for fostering a healthy and innovative environment. In the absence of an ethical framework, issues such as information plagiarism and cyberbullying may suppress students’ willingness to express themselves and hinder collaborative creativity (Le, 2023). This study found that certain variables indicative of digital responsibility—such as “spreading false information”—exhibited significant positive predictive power in the low-creativity group, suggesting a potential negative co-variation between ethical behavior and creative thinking. These findings indicate that creative thinking development requires not only technological support but also guidance rooted in values and ethical safeguards (Glăveanu et al., 2021). When creative products co-occur with problematic ICT behaviors (e.g., unverified claims, improper reuse), responses are most productive when instruction-first and restorative: separate the appraisal of creative merit from ethical compliance, require revision that includes fact-checking, proper citation/licensing, and a brief reflection note, and provide targeted supports (media-literacy micro-lessons, attribution templates). These steps are consistent with digital-citizenship and online-risk literatures that emphasize guided practice over punitive responses (Ribble, 2015), preserving creative risk-taking while upholding ethical standards. At the implementation level, a low-overhead routine—one goal-aligned digital creation task per week with rubric-based formative feedback on originality, diversity, and appropriateness—foregrounds quality over frequency and makes the effects of ICT use instructionally visible.

## 7 Conclusion and limitation

This study utilized data from the PISA 2022 ICT questionnaire and applied explainable machine learning methods to investigate the predictive mechanisms of students’ creative thinking levels. The results identified a set of key ICT interaction variables that differentiated students across creativity levels. High-creative students tended to engage more in reflective, collaborative, and expressive digital behaviors, while low-creative students often exhibited low-goal-oriented or media-unsavvy usage patterns. Several features showed nonlinear predictive trends, underscoring that the *quality* and *intentionality* of ICT use are more critical than mere *frequency* in fostering creativity.

Despite the enhanced interpretability provided by SHAP analysis, this study has limitations. First, the reliance on self-reported data may introduce subjective bias. First, this study draws on self-reported questionnaire data from East Asian PISA participants (Singapore, Hong Kong, Macao, Chinese Taipei, Korea). Region-specific cultural and instructional features may shape both ICT-use patterns and creative performance; accordingly, cross-cultural generalizations should be made prudently. Second, in terms of data processing, missing responses were handled via listwise deletion. While common in secondary analyses, this approach can bias estimates if missingness departs from complete randomness; future robustness checks may consider multiple imputation, k-nearest neighbors, or model-based ordinal imputation for Likert-type variables. For interpretability and modeling stability, we also aggregated PISA’s six creative-thinking levels into three ordered bands (low/medium/high). We acknowledge the reduced granularity that accompanies banding and therefore interpret results at the band level only, and alternative cut points or an ordinal specification could be examined in subsequent work. Finally, given the cross-sectional design, the reported relationships are associational rather than causal. Longitudinal tracking, (quasi-)experimental designs, and multi-source data (e.g., digital traces, performance tasks) would allow stronger tests of the mechanisms suggested here. These considerations delineate the scope of inference without detracting from the study’s practical value for guiding purposeful, ethically informed uses of ICT to support creative learning. It suggests that educational practices should emphasize the intentional, complex, and ethically guided use of ICT to foster higher-order creative expression and collaboration in digital environments.

## Data Availability

The datasets presented in this study can be found in online repositories. The names of the repository/repositories and accession number(s) can be found at: the original data supporting the conclusions of this article are publicly available in the PISA 2022 database at: https://www.oecd.org/pisa/data/2022database/. Derived datasets generated during the current study are available from the corresponding author on reasonable request.
